# Validation of the Chinese version of the Somatic Symptom Scale-8 in patients from tertiary hospitals in China

**DOI:** 10.3389/fpsyt.2022.940206

**Published:** 2022-09-28

**Authors:** Tao Li, Jing Wei, Kurt Fritzsche, Anne C. Toussaint, Lan Zhang, Yaoyin Zhang, Hua Chen, Heng Wu, Xiquan Ma, Wentian Li, Jie Ren, Wei Lu, Rainer Leonhart

**Affiliations:** ^1^Department of Psychological Medicine, Peking Union Medical College Hospital (CAMS), Beijing, China; ^2^Department of Psychiatry and Psychotherapy, University of Freiburg Medical Center, Freiburg, Germany; ^3^Clinic for Psychosomatic Medicine and Psychotherapy, University Medical Center Hamburg-Eppendorf, Hamburg, Germany; ^4^Mental Health Center, West China Hospital, Sichuan University, Chengdu, China; ^5^Department of Psychosomatic Medicine, Sichuan Provincial People’s Hospital, University of Electronic Science and Technology of China, Chengdu, China; ^6^Department of Psychological Medicine, Zhongshan Hospital, Fudan University, Shanghai, China; ^7^Department of Psychosomatic Medicine, Tongji Hospital, School of Medicine, Tongji University, Shanghai, China; ^8^Department of Psychosomatic Medicine, Shanghai East Hospital, School of Medicine, Tongji University, Shanghai, China; ^9^Department of Clinic Psychology, Wuhan Mental Health Center, Wuhan, China; ^10^Department of Rehabilitation, General Hospital of Jincheng Anthracite Coal Mining Group Co. Ltd., Jincheng, China; ^11^Department of Psychosomatic Medicine, Beijing Hospital of Traditional Chinese Medicine, Capital Medical University, Beijing, China; ^12^Institute of Psychology, University of Freiburg, Freiburg, Germany

**Keywords:** Somatic Symptom Scale-8, reliability, validation, psychometrics, somatic symptom disorder, Chinese version, screening tool

## Abstract

**Objective:**

To validate the Chinese language version of the Somatic Symptom Scale-8 (SSS-8) in a sample of outpatients attending tertiary hospitals in China.

**Materials and methods:**

A Chinese language version of the SSS-8 was completed by outpatients (*n* = 699) from psychosomatic medicine, gastroenterology/neurology, and traditional Chinese medicine clinics of nine tertiary hospitals between September 2016 and January 2018 to test the reliability. The Patient Health Questionnaire-15 (PHQ-15), the Somatic Symptom Disorder–B Criteria Scale (SSD-12), the Patient Health Questionnaire-9 (PHQ-9), the General Anxiety Disorder-7 (GAD-7) scale, the Medical Outcome Study 12-item Short Form Health Survey (SF-12) and the World Health Organization Disability Assessment Schedule (WHO DAS 2.0) were rated to test construct validity. The criterion validity was tested by using the Semi-structured Clinical Interview for DSM-5 (Research Version) (SCID-5-RV) for somatic symptom disorder (SSD) as the diagnostic gold standard to explore the optimal cutoff score of the SSS-8.

**Results:**

The average age of the recruited participants was 43.08 (±14.47). 61.4% of them were female. The internal consistency derived from the sample was acceptable (Cronbach α = 0.78). Confirmatory factor analyses resulted in the replication of a three-factor model (cardiopulmonary symptoms, pain symptoms, gastrointestinal and fatigue symptoms) (comparative fit index = 0.95, Tucker-Lewis index = 0.92, root mean square error of approximation = 0.10, 90% confidence interval = 0.08–0.12). The SSS-8 sum score was highly associated with PHQ-15 (*r* = 0.74, *p* < 0.001), SSD-12 (*r* = 0.64, *p* < 0.001), GAD-7 (*r* = 0.59, *p* < 0.001), and PHQ-9 (*r* = 0.69, *p* < 0.001). The patients with more severe symptoms showed worse quality of life and disability The optimal cutoff score of SSS-8 was 9 (sensitivity = 0.67, specificity = 0.68).

**Conclusion:**

Our preliminary assessment suggests that the Chinese language version of the SSS-8 has reliability and validity sufficient to warrant testing further in research and clinical settings.

## Introduction

Somatic symptoms are an important, subjective health-related experience and the most common reason for people seeking medical services (1). Somatic symptoms may be related to physical disease, but may also be a feature of a mental disorder, according to the classical medical concept of dualism. In recent decades, somatic symptoms have been considered a psychosomatic phenomenon. Somatic symptoms act as a precise key that can help doctors gain a profound understanding of a patient’s suffering and identify health problems that need intervention. From the perspective of the clinical practice of mental health, distressing somatic symptoms are an important diagnostic criterion for somatic symptom disorder (SSD) in the Diagnostic and Statistical Manual of Mental Disorders (Fifth Edition) (DSM-5) and bodily distress disorder in the International Classification of Diseases (11th Revision) (ICD-11), and are significantly correlated with patients’ quality of life.

Many scales have been designed to screen for somatic symptoms in daily clinical practice and epidemiological investigation (2). Previously, somatic symptoms were often included as part of general psychopathology measures, such as the 12 items in the Symptom Checklist-90 (SCL-90) somatization subscale (3). Since the 1980s, specific somatic symptom scales have been developed for the diagnosis of somatoform disorders, such as the Patient Health Questionaire-15 (PHQ-15) (4). Their common criteria include pain in different parts of the body, fatigue, dizziness, gastrointestinal discomfort, and breathing difficulties. These symptoms, which are the most common somatic symptoms, constitute the main content of the Somatic Symptom Scale-8 (SSS-8) as the short form of the PHQ-15 (5). In addition, sleep problems in the PHQ-15 are also retained in the SSS-8 (6).

In DSM-5 Cross Cutting Symptom Measure field trials, the SSS-8 was used as a reference measure for facilitating the diagnosis of SSD (7). Validated versions of the SSS-8 have been published in Germany, Japan, Korea, and Greece (6, 8–10). The SSS-8 has been recommended as a reliable and valid self-report measure of somatic symptom burden in the general population.

The aims of this study were (1) to investigate the reliability and validity of the Chinese version of SSS-8 in patients from tertiary hospitals in China, and (2) to explore the cutoff point of the sum score of SSS-8 to make it a valid screening tool for SSD.

## Materials and methods

### Sampling strategy

This study was a multicenter cross-sectional survey of somatic burden and the related mental well-being of outpatients attending tertiary hospitals in China between September 2016 and January 2018. Tertiary hospitals in China include general hospitals and specialized hospitals which provide advanced health care to patients. In most previous studies, the SSS-8 was proved to be a validate tool in general population (6, 8, 9). In this study, outpatients of tertiary hospitals were chosen to investigate if the SSS-8 is a validate tool for patients with relatively high somatic symptom burden. Based on geographic diversity, nine tertiary hospitals in Beijing, Shanghai, Chengdu, Wuhan, and Jincheng were selected, representing northern, southeastern, and southwestern regions of China. Outpatients were recruited from three different settings: psychosomatic medicine, gastroenterology/neurology, and traditional Chinese medicine. The neurology and gastroenterology departments were chosen to represent modern biomedical settings. In randomly selected work hours, the research assistant came to these departments’ outpatient clinics and invited all patients who came during that time to participate in the study by oral invitation. Eligible patients had to be aged 18 years or older, be seeking treatment voluntarily, and be able to read and sign the informed consent. People visiting for another person’s problems, patients with difficulty communicating, a language barrier, or limited literacy; patients with cognitive impairment, an organic brain disorder or dementia, or psychosis; and patients with acute suicidal tendencies were excluded. All eligible patients were registered, including those who, owing to lack of time, lack of interest, or lack of trust in the researchers, did not participate.

### Study procedure

Participants were asked to conduct self-rated questionnaires and an in-person interview on the spot and no extra visit for research was required.

The instruments administered in the study included a questionnaire on demographic characteristics, the SSS-8, the PHQ-15, the Patient Health Questionnaire-9 (PHQ-9), the General Anxiety Disorder-7 (GAD-7) scale, the Somatic Symptom Disorder–B Criteria Scale (SSD-12), the Medical Outcome Study 12-item Short Form Health Survey (SF-12), and the short-form self-administered World Health Organization Disability Assessment Schedule 2.0 (WHO-DAS 2.0). The Semi-structured Clinical Interview for DSM-5 (research version; SCID-5-RV) was used to conduct a diagnostic interview of each patient, and the results were used as the gold standard for SSD diagnosis, to measure the criterion validity of SSS-8.

All the questionnaires were print on papers with instructions in fixed order and filled by patients. The interview was conducted by research assistants from each tertiary hospital (students of psychology at the master’s level, students of medicine in their final year of studies, and medical doctors) trained by experienced psychiatrists. They were blinded to the patients’ questionnaire results.

### Development of the Chinese version of the Somatic Symptom Scale-8

The SSS-8 scale has eight items, each of which is divided into five categories (0 = not at all to 4 = very much). Total score is between 0 and 32 points. The validated English version of the SSS-8 has been published in 2014 (6). The questionnaire was translated and then back-translated from English into Mandarin Chinese, using the “ITC-Test Adaptation Guidelines” of the International Test Commission. Three Mandarin Chinese native speakers fluent in both written and spoken English (one psychiatrist, one psychologist, and one educator) first completed independent translations. These preliminary translations were discussed during project meetings. A pilot of the revised questionnaire was tested on 10 patients and then back-translated into English for a final revision, from which the final version of the Chinese SSS-8 was established.

### Other psychological measurements

The PHQ-15 is one of the most frequently used self-administered questionnaires, with 15 items assessing the burden of common somatic symptoms within the last 4 weeks (4). Each of the 15 items is scored on a three-point Likert-type scale with sum scores ranging from 0 to 30. A higher score indicates a heavier burden of somatic symptoms. The validity of the Chinese version was previously proven satisfactory (11).

The PHQ-9 is another widely used self-administered instrument and comprises nine items assessing depression symptoms within the last 2 weeks (12). Items are also scored on a four-point Likert-type scale with sum scores ranging from 0 to 27. Higher scores indicate more severe symptoms. The Chinese version of the PHQ-9 has demonstrated good validity in Chinese general hospital outpatients (13).

The GAD-7 self-administered questionnaire contains seven items measuring symptoms of general anxiety disorder and other common anxiety disorders (14). The items are scored on four-point Likert-type scale, and sum scores range between 0 and 21. The Chinese version of the GAD-7 has demonstrated satisfactory reliability and validity (15).

The SSD-12 consists of 12 self-administered items scored on a five-point Likert-type scale, and it assesses patients’ perceptions of the symptom-related thoughts, feelings, and behaviors that they experience, based directly on the DSM-5 criteria (16). The total score is between 0 and 48 points and a higher score indicates greater psychological distress associated with somatic symptoms. The SSD-12 Chinese version has demonstrated satisfactory validity (17).

The SF-12 measures health-related quality of life (18). Twelve self-administered items are divided into six items reflecting a physical component (physical component score of SF-12, PCS of SF-12) and six items reflecting a mental component (mental component score of SF-12, MCS of SF-12), normalized to two subscales (mean [SD] = 50 [10]). Better health status is indicated by a higher score and the Chinese version has demonstrated good validity and reliability (19).

The WHO-DAS 2.0 comprises 12 self-administered items measuring disability and health at the population level and in clinical practice (20). A five-point Likert-type scale is used for scoring each item. The simple scoring method gives a total score of between 12 and 60 points. Higher scores indicate more severe disability, and a Chinese version has been validated (21).

The SCID-5-RV for SSD has previously been translated according to the procedures described for the translation of the SSS-8. The validity of the Chinese version in assessing for SSD has been demonstrated (22).

### Statistical analysis

SPSS version 25 was used for data analysis except factor analysis. The internal consistency of the scale was assessed with Cronbach’s Alpha coefficients. Split-half reliability was estimated using Spearman-Brown coefficients.

Mplus 8.2 software was used for factor analysis to test the factorial structure of the SSS-8 for the Chinese samples. To test global model fits, comparative fit index (CFI), Tucker-Lewis index (TLI), and the root mean square error of approximation (RMSEA) were used. A CFI > 0.95, TLI > 0.95, and RMSEA < 0.08 were taken to indicate a good fit for the continuous data (23).

Pearson correlations coefficients were calculated to correlate the sum score of the SSS-8 with the PHQ-15, PHQ-9, GAD-7, and SSD-12, which assess construct validity of the SSS-8.

Receiver operating characteristic curve (ROC) was used to examine the effectiveness of the SSS-8 as a screening tool for SSD. The Youden Index was used to choose the optimal threshold value (cutoff point) of the SSS-8 for which the diagnostic specificity + sensitivity-1 is maximized for SSD (24).

The severity categories, general disability (WHO-DAS 2.0), the physical component score of SF-12 and the mental component score of SF-12 were analyzed by one-way analysis of variance followed by multiple comparisons using Scheffe test or Dunnett’s T3 test according to the homogeneity state of variances.

Floor and ceiling effects were considered to be present if more than 60% of subjects achieved the lowest or highest score on each item and the sum score of the SSS-8 (8).

## Results

**FIGURE 1:**
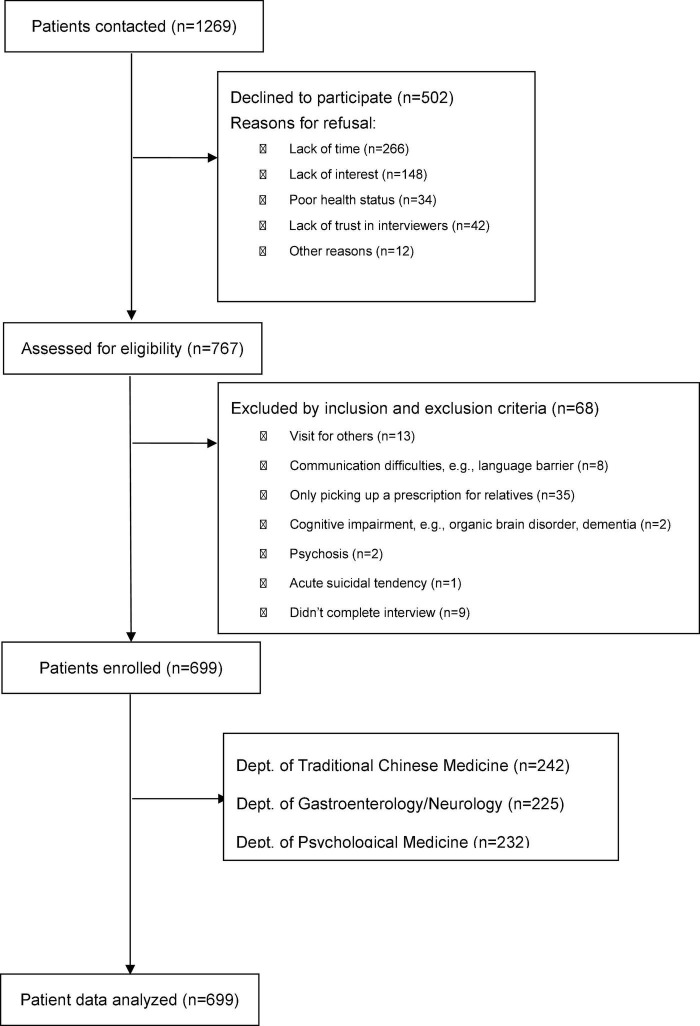
Flow chart of patient enrolment.

A total of 1,269 patients were contacted, of whom 699 (55.1%) were enrolled (Figure 1). Sixty-eight (5.4%) patients were excluded according to the exclusion criteria, and 502 (39.6%) patients refused to participate in the study. Of those patients who refused, 53% indicated that they did not have time to participate, 29.5% were not interested in participating, and 8.4% indicated distrust of the researchers. A further 6.8% felt too unwell to participate and 2.4% gave other reasons. Table 1 indicates the sociodemographic characteristics of the sample.

**TABLE 1:** Sociodemographic characteristics of outpatient sample from general hospitals in China.

	Total (*n* = 699)	With SSD (*n* = 236*)	Without SSD (*n* = 461*)	t/Chi^2^	df	*P*
				
	Mean (SD) or N (%)	Mean (SD) or N (%)	Mean (SD) or N (%)			
Age	43.08 (14.47)	42.99 (14.04)	43.05 (14.69)	0.05	695	0.957
Sex (female)	429 (61.4)	143 (60.6)	284 (61.6)	0.07	1	0.795
Ethic group (Han)	650 (93.0)	219 (92.8)	429 (93.1)	0.02	1	0.898
Health insurance	602 (86.1)	200 (84.7)	400 (86.8)	0.69	1	0.406
Residence (city)	575 (82.3)	187 (79.2)	386 (83.7)	1.85	1	0.174
Marital status (married)	512 (73.3)	163 (69.1)	343 (74.4)	2.23	1	0.135
Monthly family income				3.26	2	0.196
Less than 4,000 RMB	235 (33.6)	89 (37.7)	145 (31.5)			
4,000–8,000 RMB	242 (34.6)	80 (33.9)	162 (35.1)			
More than 8,000 RMB	217 (31.0)	65 (27.5)	151 (32.8)			
Unknown	5 (0.7)	2 (0.8)	3 (0.7)			
Education level				2.16	2	0.340
Elementary	45 (6.4)	18 (7.6)	27 (5.8)			
Middle school	318 (45.5)	113 (47.9)	204 (44.2)			
University or higher	336 (48.1)	105 (44.5)	230 (49.9)			
Occupation				5.61	3	0.132
Employed/Student	382 (54.7)	119 (50.4)	262 (56.8)			
Unemployed	130 (18.6)	39 (16.5)	47 (10.2)			
Retired	150 (12.3)	51 (21.6)	98 (21.2)			
Others	37 (5.3)	27 (11.4)	54 (11.7)			
Department				34.05	2	<0.001
Biomedical settings	225 (32.2)	90 (38.1)	134 (29.1)			
Traditional medicine settings	232 (33.2)	44 (18.6)	187 (40.6)			
Psychosomatic medical settings	242 (34.6)	102 (43.2)	140 (30.4)			
Sum score of scales						
SSS-8	8.70 (6.08)	11.97 (6.50)	7.03 (5.09)	10.18	386.05	<0.001
PHQ-15	9.34 (5.40)	12.01 (5.53)	7.96 (4.77)	9.54	415.15	<0.001
SSD12	13.98 (12.23)	23.60 (11.43)	9.06 (9.38)	16.84	400.50	<0.001
PHQ-9	8.48 (6.59)	11.84 (6.76)	6.75 (5.78)	9.85	411.59	<0.001
GAD-7	6.64 (5.89)	9.70 (6.08)	5.09 (5.15)	9.94	408.48	<0.001
Physical component score of SF-12	43.07 (9.20)	39.14 (8.98)	45.14 (8.57)	8.58	691	<0.001
Mental component score of SF-12	41.35 (12.34)	34.86 (11.30)	44.64 (11.54)	10.63	691	<0.001
WHO-DAS 2.0 (SD)	19.08 (7.28)	22.65 (8.52)	17.25 (5.78)	8.75	346.93	<0.001

SSD, somatic symptom disorder; SD, Standard deviation; SSS-8, Somatic Symptom Scale-8; PHQ-15, The Patient Health Questionnaire-15; SSD-12, the Somatic Symptom Disorder–B Criteria Scal; PHQ-9, the Patient Health Questionnaire-9; GAD-7, the General Anxiety Disorder-7; SF-12, the Medical Outcome Study 12-item Short Form Health Survey; WHO DAS 2.0, the World Health Organization Disability Assessment Schedule. *2 of 699 participants had incomplete diagnostic information.

225 (32.2%) patients were enrolled from biomedical settings, 90 (40.0%) of which were SSD positive. 232 (33.2%) patients were enrolled from Traditional Chinese Medical settings, 44 (19.0%) of which were SSD positive. 242 (34.6%) patients were from psychosomatic medical settings, 102 (42.1%) of which were SSD positive. The prevalence of SSD in patients from different medical settings were significantly different (χ^2^ = 34.049, df = 2, *p* ≤ 0.001).

### Reliability of the Somatic Symptom Scale-8

The SSS-8 demonstrated an acceptable reliability for the sample, with Cronbach α = 0.78. Additionally, Spearman-Brown split-half coefficient was 0.73. Table 2 shows the overall item and subscale characteristics. The sum scores for 26 out of 699 participants were 0, for 1 out of 699 participants was 32. No remarkable floor or ceiling effects were observed for the total scores or individual item scores.

**TABLE 2:** Frequency distribution of responses (%), mean (SD), and item-total correlations for the items of the SSS-8.

Item	Not at all	A little bit	Some-what	Quite a bit	Very much	Mean (SD)	Cor_iT_	Cron.α_id_
1. Stomach or bowel problems	34.5	26.8	19.2	14	5.4	1.29 (1.23)	0.31	0.79
2. Back pain	56.8	22.2	11	6.2	3.9	0.78 (1.11)	0.48	0.76
3. Pain in your arms, legs, or joints	51.2	24.7	13.4	7	3.6	0.87 (1.11)	0.42	0.77
4. Headaches	52.3	23.1	11.6	9	4	0.89 (1.16)	0.55	0.75
5. Chest pain or shortness of breath	51.9	24.6	12.7	7.9	2.9	0.85 (1.10)	0.56	0.75
6. Dizziness	50.5	25	12	8.3	4.1	0.91 (1.15)	0.49	0.76
7. Feeling tired or having low energy	25.8	28.3	20.5	16.3	9.2	1.55 (1.28)	0.64	0.73
8. Trouble sleeping	31.9	22.3	16.5	16.9	12.4	1.56 (1.40)	0.47	0.76

Cor_iT_, Corrected item-total correlation; Cron. α_id_, Cronbach α if item deleted; SD, standard deviation.

### Validity of the Somatic Symptom Scale-8

#### Factorial validity

Previous studies have carried out factor analysis of the SSS-8. Confirmatory factor analysis (CFA) was adopted in our study. Three factor models were verified: a general-factor model, a three-factor model, and a second-order factor model that contained a global factor and four low-grade symptom clusters (6, 9). Fit indices for the three models from the CFA of the total included patients (*n* = 699) are shown in Table 3 (see attachment). The relatively better fit indices were those derived from the three-factor model (Tucker-Lewis index, 0.92; CFI, 0.95; root mean square error of approximation, 0.10, 90% confidence interval [CI]: 0.08–0.12). The three-factor model consisted of three symptom clusters: (1) cardiopulmonary symptoms (Cronbach α = 0.71) including headaches (item 4), chest pain or shortness of breath (item 5), and dizziness (item 6); (2) pain symptoms (Cronbach α = 0.64) including back pain (item2) and painful legs, arms, or joints (item 3); and (3) gastrointestinal and fatigue symptoms (Cronbach α = 0.57) including bowel or stomach problems (item 1), tiredness or having low energy (item 7), and sleeping difficulties (item 8).

**TABLE 3:** Fit indices for three SSS-8 models from confirmatory factor analysis of total patients (*n* = 699).

	General-factor model	Three-factor model			Second-order factor model			
			
		Cardiopulmonary symptoms	Pain symptoms	Gastrointestinal and fatigue symptoms	General factor			
	
					Gastrointestinal	Pain	Cardiopulmonary	Fatigue
Loading								
Item 1	0.38**			0.40**	0.38**			
Item 2	0.61**		0.79**			0.69**		
Item 3	0.57**		0.71**			0.58**		
Item 4	0.73**	0.77**				0.74**		
Item 5	0.70**	0.74**					0.70**	
Item 6	0.70**	0.73**					0.71**	
Item 7	0.76**			0.83**				0.84**
Item 8	0.57**			0.60**				0.62**
Factor correlations								
Cardiopulmonary symptoms		1	0.63	0.85				
Pain symptoms		0.63	1	0.68				
Gastrointestinal and fatigue symptoms		0.85	0.68	1				
Model fits								
χ^2^ (df)	245.27 (20)	132.32 (17)			218.86 (19)			
TLI	0.87	0.92			0.88			
CFI	0.91	0.95			0.92			
RMSEA (90% CI)	0.13 (0.11–0.14)	0.10 (0.08–0.12)			0.12 (0.11–0.14)			

SSS-8, Somatic Symptom Scale-8; TLI, Tucker-Lewis index; CFI, comparative fit index; RMSEA, the root mean square error of approximation; CI, confidence interval. **P < 0.001.

Meanwhile, the fit indices for the general-factor model (TLI, 0.87; CFI, 0.91; RMSEA, 0.13, 90% CI: 0.11–0.14) and for the second-order factor model (TLI, 0.88; CFI, 0.92; RMSEA, 0.12, 90% CI: 0.11–0.14) were rejected.

#### Criterion validity

**FIGURE 2:**
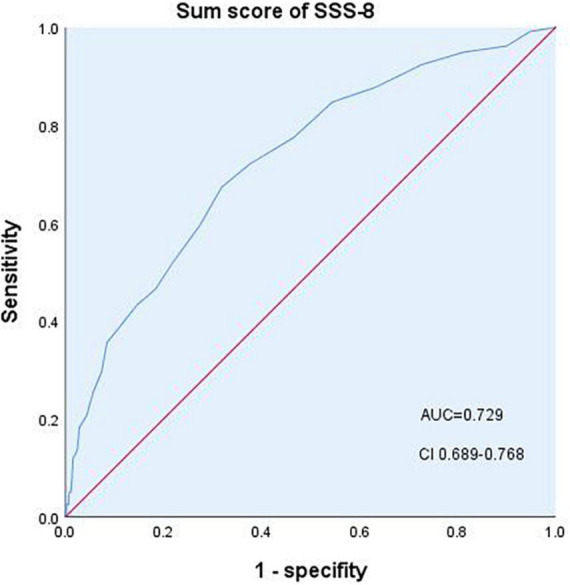
Diagnostic performance of the SSS-8 sum score. AUC, area under the curve; CI, confidence interval.

SSD diagnosed using the SCID-5-RV was used as the gold standard to test the criterion validity and threshold of the SSS-8. A total of 697 participants completed the interview. Of these, 236 (33.9%) participants were diagnosed with SSD. The diagnostic effectiveness of the SSS-8 for SSD was tested by using the sum score of the SSS-8 (Figure 2; Table 4). Area under curve (AUC) for detecting SSD was 0.729. The best diagnostic performance of the SSS-8 sum score was achieved with a cutoff of ≥9 in the total sample (Youden index = 0.355, sensitivity = 0.674, and specificity = 0.681), with 0.520 positive predictive value (PPV) and 0.803 negative predictive value (NPV).

**TABLE 4:** Result of the ROC analysis of the SSS-8 sum score for the SSD diagnosis.

Cutoff	Sensitivity	Specificity	Youden Index	PPV**	NPV**
9*	0.674	0.681	0.355	0.520	0.803
4	0.924	0.273	0.197	0.394	0.875
8	0.720	0.625	0.345	0.496	0.814
12	0.466	0.816	0.282	0.564	0.749
16	0.297	0.926	0.223	0.730	0.745

ROC analysis: Receiver Operating Characteristic analysis; SSS-8, Somatic Symptom Scale-8; SSD, somatic symptom disorder; PPV, positive predictive value; NPV, negative predictive value. *Cut off score with the highest Youden’s index value. **PPV and NPV was calculated based on the prevalence from the research data.

In previous studies, five categories of severity were defined according to SSS-8 sum scores of 0–3 (no to minimal severity), 4–7 (low), 8–11 (medium), 12–15 (high), and ≥16 (very high). Sensitivity and specifificity were calculated with each cut-off score. Detailed results are presented in Table 4.

#### Construct validity

The various well-established scales were used to assess construct validity of the SSS-8 Chinese version. The mean (SD) score for the PHQ-15 (*n* = 696) was 9.34 (5.40), which correlated highly with the SSS-8 sum score (*r* = 0.74, *p* < 0.001). Moderate to high correlations were also found between the SSS-8 sum score and symptoms associated anxiety (SSD-12: *r* = 0.61, *p* < 0.001), general anxiety symptoms (GAD-7: *r* = 0.56, *p* < 0.001), depression symptoms (PHQ-9: *r* = 0.67, *p* < 0.001), health-related quality of life (PCS of SF-12: *r* = –0.48, *p* < 0.001; MCS of SF-12: *r* = –0.52, *p* < 0.001), and health-related disability (WHO DAS 2.0: *r* = 0.55, *p* < 0.001) as expected. The mean (SD) scores of the SSD-12, GAD-7, PHQ-9, PCS of SF-12, MCS of SF-12 and WHO DAS 2.0 were 13.98 (12.23), 6.64 (6.59), 8.48 (6.59), 43.07 (9.20), 41.35 (12.34) and 19.08 (7.28) respectively (Table 1).

#### Severity categories

The health-related quality of life and general disability of patients were caculated respectively based on the severity of categories of SSS-8 (6). The patients with more severe symptoms showed worse quality of life and disability. One-way analysis of variance followed by multiple comparisons using Scheffe test for the the mental component score of SF-12 and Dunnett’s T3 test for the physical component score of SF-12 and the WHO-DAS 2.0 sum score was performed. Significant differences was observed between all pairs, except for the pair of the medium group and the high group (Table 5).

**TABLE 5:** Quality of life and disability according to SSS-8 severity category in tertiary hospital patients in China.

SSS-8 severity category (Range)	Number of patients, *n* (%)	SSD diagnosis, *n* (%)	Physical component score of SF-12 (SD)	Mental component score of SF-12 (SD)	WHO-DAS 2.0 (SD)
No to minimal (0–3)	144 (20.7)	18 (12.4%)	49.39 (7.06)	50.16 (9.95)	14.63 (3.88)
Low (4–7)	210 (30.3)	48 (22.9%)	44.96 (8.01)	44.74 (10.26)	17.02 (4.98)
Medium (8–11)	147 (21.2)	60 (40.5%)	41.06 (8.83)	38.98 (11.48)	19.70 (6.79)
High (12–15)	90 (13.0)	40 (44.0%)	40.57 (7.43)	34.85 (10.76)	21.65 (6.82)
Very high (≥16)	103 (14.8)	70 (66.7%)	35.43 (8.95)	31.20 (10.41)	26.22 (9.25)

SSS-8, Somatic Symptom Scale-8; SSD, somatic symptom disorder; SF-12, the Medical Outcome Study 12-item Short Form Health Survey; WHO DAS 2.0, the World Health Organization Disability Assessment Schedule; SD, Standard deviation.

## Discussion

In the sample of our cross-sectional multicenter research, the SSS-8 demonstrated acceptable reliability (Cronbach α = 0.78). The confirmatory factor analysis shows that 3-factor model can be acceptable in our sample. The sum score for the SSS-8 was moderately to highly correlated with the PHQ-15, PHQ-9, GAD-7, SF-12, and WHO-DAS 2.0. This is consistent with previous studies and expectations (17, 25–27). The optimal cutoff score for SSD is 9 with sensitivity 0.674 and specificity 0.681. The severity category criteria set by Gierk (6) were valid in our Chinese language version of the SSS-8.

In psychometrics researches, the sampling range will have an important impact on the results. Previously, there were studies based on general population and study based on patient from psychosomatic department which had different baseline of somatic symptom burden (6, 8, 28). In Gierk’s study, the mean score of SSS-8 was 3.23 (3.96). In Matsudaira’s study, the mean score of SSS-8 was 4.5 (5.2). In Toussaint’s study, the mean score of SSS-8 was 13.3 (5.6). We focused on outpatients from different medical settings of tertiary hospital, which were not only enrolled from psychosomatic clinics and biomedical settings with relatively high prevalence of SSD like gastroenterology department and neurology department, but also from traditional Chinese medicine department. The mean score of the SSS-8 in our study was 8.70 (6.08), which was in between the general population and patients from psychosomatic department.

We did not observe a significant floor or ceiling effect for any of the SSS-8 items, which can be observed in general population investigations and in patients from specific clinics (8, 10, 28).

In this study, the fit indices of all three models were generally worse than previous studies, and can’t fully fit the criteria (CFI > 0.95, TLI > 0.95, and RMSEA < 0.08). In Gierk’s research, the second-order factor model showed good fit indices (TLI, 0.95; CFI, 0.97; RMSEA, 0.08 [90% CI, 0.08–0.09]) (6). The fit indices of the general factor model in the same reseach were less ideal (TLI, 0.91; CFI, 0.94; RMSEA, 0.11 [90% CI, 0.10–0.12]). In Yang’s research, 3-factor model was tested and showed excellent fit indices (TLI, 1.022; CFI, 1.000; RMSEA, 0.00) (9). It is possible that the selection of participants affected the distribution of symptoms. Nearly one third participants were recruited from gastrointestinal department and neurology department who could have specific symptoms like stomach or bowel problems; headache; and dizziness. Besides, the influence of cultural background on symptom distribution cannot be excluded.

The SSS-8 used as screening tools for SSD shows moderate diagnostic accuracy (AUC = 0.729). Similar conclusion was proved by Toussaint et al. (AUC = 0.71) (28). But the cutoff points are different: 12 in Toussaint’s study, 9 in this study. As we mentioned above, the difference in the overall level of somatic symptom burden between the two groups of patient may influence the level of the cut-off value.

In Toussaint’s study, the sensitivity and specificity (72 and 59%) were also similar with the results of our study. It came to the same conclusion that the efficiency of SSS-8 as a single tool for screening SSD was not very satisfactory, especially in patients with relatively high somatic symptom burden. The screening efficiency of SSS-8 for SSD in the general population remains to be explored in the future.

While the SSS-8 focused on the A-criteria of SSD, there is other scale be used to screen SSD, like SSD-12 which is focused on the B-criteria of SSD. Based on the same sample of patients in this study, the criteria validity was tested with SSD-12. The SSD-12 showed higher criterion validity (AUC = 0.837) for SSD (17). According to our data, scale focus on B-criteria of SSD like SSD-12 may be more powerful for screening SSD than scale focus on A-criteria (SSS-8). In the sample of toussaint’s study which was from the outpatient clinic of the department of Psychosomatic Medicine and Psychotherapy of the University Medical Center Hamburg, Germany, 86.8% of the participants fulfilled the A criterion of SSD, whereas a total of 56.2% met the full DSM-5 diagnostic criteria (27). The huge gap between the percentage of patients fulfilling A-criteria and fulfilling the diagnostic criteria of SSD in DSM-5 also indicated the limitation of the screening efficacy of SSS-8 for SSD. The combination of the two kinds of scale for screening is still worth further exploration. When SSS-8 is used alone in clinical work, it is necessary to carefully explain the suggestive significance of the results for SSD diagnosis.

We followed the method and criteria for categorizing severity developed by Gierk (6). In Gierk’s study, patients with higher severity visited the hospital more frequently. In present study, patients with higher severity were found to have higher disability and worse quality of life. Due to the lack of statistically significant inter-group differences between the moderate and high severity groups, further studies are needed to determine whether the severity classification should be adjusted in Chinese patients.

Finally, the prevalence of SSD in this study population (33.9%) was relatively high (29). The results of prevalence were influenced by the sampling method. Departments selected in this study were the departments where patients with SSD most frequently visited. This would overestimate the prevalence of somatic symptom disorders among outpatients in tertiary hospitals in China.

There are several limitations of the present study. Limited departments of tertiary hospitals were selected to recruit patients. The symptom clusters of patients in these departments may have certain characteristics and cannot represent the outpatients in all departments of tertiary hospitals, which affects the distribution of symptoms and limit the generalizability of the result of this study. The high refusal rate for participation might also cause bias. Comorbid mental or physical disorders were not addressed in this study. This made it impossible to compare the difference in somatic symptom burden between patients with physical disorders and patients with SSD. However, because the diagnosis criteria of SSD in DSM-5 does not require the exclusion of physical diseases, it had little impact on the evaluation of diagnostic validity. The order of the questionnaires were fixed. The SSS-8 ranked second to last questionnaires (SF-12). The effect of sequence can’t be avoid. It should be improved in the future by vary the orders of questionnaires which will be more convenient when using online way.

In addition, it is of great significance to test the psychometrics of Chinese SSS-8 in the general population of China for exploring the influence of cultural background on somatic symptoms.

In conclusion, the Chinese language version of the SSS-8 demonstrated satisfactory reliability and validity among outpatients attending tertiary hospitals. Our results indicate that it can be used as a screening tool to assess for the burden of somatic symptoms, not only in the general population but also with hospital patients.

## Data availability statement

The original contributions presented in this study are included in the article/supplementary material, further inquiries can be directed to the corresponding author.

## Ethics statement

The studies involving human participants were reviewed and approved by University of Freiburg (no. 494/17) and Peking Union Medical College Hospital (no. S-K276). The patients/participants provided their written informed consent to participate in this study.

## Author contributions

TL was the project leader and responsible for the organization of data collection, analysis and drafted the manuscript. JW, KF, and AT contributed to the study conception and design. LZ, YZ, HC, HW, XM, WLu, JR, and WLi participated in its design and coordination and helped to draft the manuscript. RL participated in the study design and performed the statistical analysis. All authors have read and approved the final manuscript.
